# Supplementary material for: Obesity Prevention within the Early Childhood Education and Care Setting: A Systematic Review of Dietary Behavior and Physical Activity Policies and Guidelines in High Income Countries
Source: Int J Environ Res Public Health. 2021 Jan 19;18(2):838. doi: 10.3390/ijerph18020838 (PMC7835808; doi:10.3390/ijerph18020838)
Supplement: Supplementary file 1 [file ijerph-18-00838-s001.pdf]

Table S1. Online Database Search Strategy.

	Medline Search: Inception- September 2020
**1.**	(Early childhood education and care).mp.
**2.**	Kinder*.mp.
**3.**	Early education.mp.
**4.**	Preschool.mp.
**5.**	Child care/
**6.**	1 or 2 or 3 or 4 or 5
**7.**	Food/
**8.**	Nutrition.mp.
**9.**	Diet/
**10.**	Healthy eating.mp.
**11.**	Physical activity.mp.
**12.**	Exercise/
**13.**	Physical fitness/
**14.**	Screen time/
**15**	Sedentary.mp.
**16.**	Sleep/
**17.**	Obes*.mp.
**18.**	7 or 8 or 9 or 10 or 11 or 12 or 13 or 14 or 15 or 16 or 17
**19.**	Exp guideline/
**20.**	Guideline as topic
**21.**	Exp practice guideline/
**22.**	Health planning guidelines/
**23.**	(guideline or practice guideline).pt
**24.**	(position statement* or policy* statement* or practice parameter* or best practice*)ti,ab,kw
**25.**	(standards or guideline or guidelines).ti,ab,kw
**26.**	((practice) adj guideline*).ab.
**27.**	Consensus*.ti,ab,kw
**28.**	Recommendat*.ti,ab,kw
**29.**	Benchmarks.mp.
**30.**	19 or 20 or 21 or 22 or 23 or 24 or 25 or 26 or 27 or 28 or 29
**31.**	6 AND 18 AND 30

Table S2. Grey Literature Search Strategy.

**1.**	**"Early Childhood Education and Care"**
2.	Kindergarten
3.	Child*
4.	1 or 2 or 3
5.	Nutrition
6.	Food
7.	Physical*
8.	"Screen time"
9.	Sedentary
10.	Sleep
11.	Obes*
12.	5 or 6 or 7 or 8 or 9 or 10 or 11
13.	Guideline
14.	Statement
15.	Policy
16.	Recommendation
17.	Strategy
18.	13 or 14 or 15 or 16 or 17
19.	4 and 12 and 18

Table S3. Guideline AGREE II appraisal scores.

Guideline	Scope and Purpose	Stakeholder Involvement	Rigour of Development	Clarity of Presentation	Applicability	Editorial Independence	Overall Assessment
Ten ways to empower children to live healthy lives: standards to empower child care facilities in Arizona (3rd Ed) (USA)	86%	48%	39%	57%	57%	57%	Recommended with modification
Action Plan for Preventing Child and Adolescent Obesity. Promoting Healthy Lifestyles and Preventing Obesity In the Child Care setting (USA)	50%	52%	34%	36%	29%	36%	Not recommended
Active Early: a Winconsin guide for improving childhood physical activity (2nd Ed) (USA)	93%	86%	57%	100%	86%	86%	Recommended with modification
Healthy Bites: a Winconsin guide for improving childhood physical activity (2nd Ed) (USA)	93%	95%	57%	93%	93%	71%	Recommended with modification
Australian 24-hour movement guidelines for the early years (birth to 5 years) (Australia)	100%	71%	43%	93%	36%	100%	Recommended with modification
Position of the Academy of Nutrition and Dietetics: Benchmarks for Nutrition in Childcare (USA)	100%	90%	52%	57%	71%	100%	Recommended with modification
Best Practices for Healthy Eating: a guide to help children grow up healthy (USA)	93%	57%	48%	100%	43%	36%	Recommended with modification
Best Practices for Physical Activity: for organizations serving children and youth a guide to help children grow up healthy (USA)	100%	57%	50%	100%	43%	36%	Recommended with modification
Canadian 24-hour movement guidelines for the early years (0-4 years): an integration of physical activity, sedentary behaviour and sleep (Canada)	100%	100%	100%	100%	100%	100%	Recommended without modification
Caring for Children (Australia)	93%	86%	43%	86%	79%	50%	Recommended with modification
Caring for our children, National Health and Safety Performance Standards: Guidelines for Early Care and Education Programs (USA)	93%	81%	71%	100%	93%	57%	Recommended without modification
Early child care obesity prevention recommendations (USA)	86%	48%	45%	100%	39%	36%	Recommended with modification

Early childhood obesity prevention policies (USA)	93%	76%	68%	86%	71%	64%	Recommended without modification
Eat Better Start Better: Voluntary Food and Drink Guidelines for Early Years Setting in England – a Practical Guide (England)	100%	71%	46%	100%	79%	57%	Recommended with modification
Eating Well for Under-5s in Child Care: Practical and nutritional guidelines, 2nd edition (UK)	100%	86%	46%	93%	75%	43%	Recommended with modification
Enacting eight policies to prevent childhood obesity: projected outcomes for Louisiana (USA)	100%	43%	38%	100%	32%	57%	Recommended with modification
Food and Nutrition Guidelines for Pre-school services (Ireland)	100%	86%	41%	57%	61%	43%	Recommended with modification
Food&Nutrition for childcare settings: Best Practice guidance (Wales)	100%	71%	46%	79%	79%	43%	Recommended with modification
Get Up & Grow: Healthy Eating and Physical Activity for Early Childhood (Australia)	100%	81%	38%	100%	82%	43%	Recommended with modification
Menu Planning in Childcare (Australia)	71%	52%	46%	86%	75%	71%	Recommended with modification
Long Day Care Menu Planner (Australia)	57%	62%	25%	86%	57%	50%	Recommended with modification
Start Right, Eat Right (Australia)	100%	57%	34%	93%	75%	57%	Recommended with modification
Healthy Food and Drink Guidance- Early Learning Services (NZ)	100%	62%	43%	86%	25%	57%	Recommended with modification
Healthy Kids, Healthy Future (USA)	100%	57%	54%	100%	46%	57%	Recommended with modification
Improving healthy weight in children: The healthiest next generation initiative (USA)	93%	90%	54%	93%	39%	57%	Recommended with modification
Let's Go! Health Care Toolkit (USA)	71%	71%	48%	50%	68%	71%	Recommended with modification
Menu planning guidelines for long day care (Australia)	93%	62%	45%	93%	68%	57%	Recommended with modification
Missouri Move Smart Child Care (USA)	86%	76%	34%	100%	75%	36%	Recommended with modification

Model Child care health policies (5th Edition) (USA)	86%	86%	43%	57%	82%	79%	Recommended with modification
Munch & Move (Australia)	100%	90%	52%	100%	89%	79%	Recommended with modification
Nutrition and Physical Activity Best Practices for Child Care Centers (USA)	86%	48%	39%	86%	18%	57%	Recommended with modification
Nutritional Guidance for early years food choices for children aged 1-5 years in early education and child-care settings (Scotland)	100%	100%	52%	86%	64%	71%	Recommended with modification
Nutrition and Wellness Tips for Young Children: provider handbook for the child and adult care food program (USA)	100%	76%	34%	100%	75%	64%	Recommended with modification
Right Bite, Easy Guide to Healthy Food and Drink Supply for South Australian Schools and Preschools (Australia)	100%	95%	39%	64%	89%	57%	Recommended with modification
Setting the Table- Nutritional Guidance and Food Standards for Early Years Childcare Providers in Scotland (Scotland)	100%	67%	55%	93%	57%	14%	Recommended with modification
Sit less, move more, sleep well: Active play guide-lines for under-fives (NZ)	100%	71%	55%	93%	50%	36%	Recommended with modification
The preschool initiative: building a healthy founda-tion for life (USA)	100%	76%	39%	57%	75%	43%	Recommended with modification
Wellness Guidelines for Alaska's Young Children: A toolkit for child care providers and families (USA)	100%	57%	45%	100%	68%	36%	Recommended with modification
